# Supplementary material for: Underwater acoustic target recognition method based on a joint neural network
Source: PLoS One. 2022 Apr 29;17(4):e0266425. doi: 10.1371/journal.pone.0266425 (PMC9053803; doi:10.1371/journal.pone.0266425)
Supplement: Attachment — Submitted filename: Response to Reviewers.docx [file pone.0266425.s001.docx]

**Paper Title:** Underwater Acoustic Target Recognition Method Based on

Joint Neural Network

**Authors:** Xingcheng Han, Chenxi Ren, Liming Wang, Yunjiao Bai

**Manuscript Number:** PONE-D-21-38631

**Date:** February 10, 2022

**Response to the Reviewers**

We would like to thank the reviewers and the editor for their time in reviewing our paper and suggestions for improving it. In this document we have written a detailed point-by-point response to the reviewers to address their comments and explain the corresponding changes made in the manuscript. Throughout this document, we use the following conventions:

- *Blue text in Italics* = Reviewers’ comments.
- Red text = New additions to the manuscript.
- Black text = Authors’ responses.

This response letter is a self-contained document that conveniently includes all the revisions.

We hope that the current revision meets your approval.

Kind regards,

Xingcheng Han, Chenxi Ren, Liming Wang, Yunjiao Bai

We would like to thank the reviewers for their valuable comments, which have helped us to further improve the contribution of the paper. We have addressed all the comments and suggestions and revised our manuscript as follows:

**For Reviewer: 1**

**Response to the comments**

**Comment 1:** *The language is unclear in many places, making it difficult to understand. Moreover, there are several grammatical mistakes. Many sentences are poorly constructed such as the first line of Abstract, a paragraph in Section 2.1 starting with “Due to extract the characteristics……...average pooling”, a paragraph in Section 3.2 starting with “The first is to extract……..eigenvector of (128,1)”, the first paragraph of Conclusion etc. Such mistakes can be noticed throughout the manuscript. So, it is highly recommended that the authors perform a thorough proofread before submitting the manuscript.*

**Our Response:** The authors thank the reviewer for this comment. According to the reviewer's suggestion, the authors have reorganized and proofread the manuscript.

**Comment 2:** *In various places, the authors claim that the joint network is proposed for the first time. There are several articles that utilize combined CNN and LSTM in underwater target recognition; the only difference here is that instead of 2D CNN, the authors make use of 1D CNN. So, it will be better to avoid such writings, or else, they should specifically emphasize the novelty of the current work by focusing on 1D CNN.*

**Our Response:** The authors thank the reviewer for this comment. According to the reviewer's suggestion, the author have revised the writing and explained why 1D CNN is used for underwater acoustic target recognition in this manuscript.

**Comment 3:** *The authors need to significantly improve the technicality of the manuscript. The contents related to underwater target recognition and network architecture should be more technical. In many places, descriptions are provided in a casual manner. It is recommended to refer to good articles published in these domains to get a good technical background.*

**Our Response:** The authors thank the reviewer for this comment. According to the reviewer's suggestion, the authors have reorganized and proofread the content related to underwater target recognition and network architecture, as well as the entire manuscript, to ensure that the manuscript is more technical.

**1.Introduction**
**Comment 1:** *The authors need to properly establish why they have come up with such an architecture. In this context, they must elaborate on the shortcomings/drawbacks of the existing models in underwater target recognition, such as joint architectures of 2D CNN and LSTM as well as 1D CNN and LSTM employing filter bank learning from raw acoustic inputs. The Introduction section need to be reframed accordingly to include a concise literature survey on these topics and a proper justification of the proposed architecture.*

**Our Response:** The authors thank the reviewer for this comment. According to the reviewer's suggestion, the authors expound the respective characteristics of 1D CNN and 2D CNN in underwater target recognition, as well as the specific reasons why 1D CNN is used for underwater acoustic target recognition.

**Actions on Manuscript:** In the fifth paragraph of the introduction, the following sentences have been added to expand the manuscript text and improve the quality of the paper:

Because the recognition framework of a single neural network makes the extraction of all features of underwater acoustic signals challenging [18-20], the research is usually focused on the development of deeper and more complex networks [21-29], which however are more difficult to train (in terms of training data size and labeling requirements). Therefore, building a new network model by combining various network structures may be a good solution. Studies on joint neural networks are mostly based on traditional two-dimensional (2D) CNN and LSTM. However, owing to the characteristics of the network model, 2D CNN seem to perform better in the field of image recognition. Conversely, 1D CNN are usually employed in speech-processing fields such as sequence modeling and natural language processing, where the use of 1D CNN reduce the amount of computation required. Therefore, in this study, a 1D CNN and LSTM network model are combined to identify underwater acoustic targets, with the aim to obtain a network with a higher training speed and recognition rate.

**Comment 2:***It seems a good practice to provide citations to works or use et al (with reference) rather than mentioning the full affiliations of the authors (as can be seen in the third and fourth paragraphs).*

**Our Response:**The authors thank the reviewer for this comment. According to the reviewer's suggestion, the authors have revised the citation style of the references and removed the affiliation of the authors about the references.

**Comment 3:** *Correct the grammatical mistakes in various places.*

**Our Response:** The authors thank the reviewer for this comment. According to the reviewer's suggestion, the authors have corrected the grammatical mistakes of the introduction section.

**2.Recognition principle**

**Comment 1:** *It will be better to reduce the theoretical part of CNN and LSTM; instead the authors may concentrate more on why CNN and LSTM have been chosen in the context of the current study. Also, it is not of much relevance to provide Fig. 1 & Fig. 2, as they are generic and well known.*

**Our Response:** The authors thank the reviewer for this comment. According to the reviewer's suggestion, in the introduction，authors explain why 1D CNN and LSTM are used to build a joint network. 1D CNN is widely used in speech fields such as sequence modeling and natural language processing, while long short-term memory (LSTM) is suitable for processing and predicting time series. However, the recognition framework of a single neural network makes the extraction of all features of underwater acoustic signals challenging, the research direction usually focused on the development of deeper and more complex networks. Therefore, building a new network model by combining various network structures may be a good solution. That is the reason why authors choose 1D CNN and LSTM to build joint network for underwater acoustic target recognition. And according to the reviewer comment 2 and comment 3 in this part, Fig.1 and Fig.2 are retained and modified based on comments made by reviewer.

**Comment 2:** *The activation function in Fig. 1 is misleading. If the authors intended to show ReLU, draw it appropriately.*

**Our Response:** The authors thank the reviewer for this comment. According to the reviewer's suggestion, the ReLU of the activation function in Fig.1 is added.

**Actions on Manuscript:** The figure1 with ReLU added to the activation function is shown below:

**Fig 1.** **1D-Convolutional neural network model.**

**Comment 3:**  *It is recommended to improve the drawing quality of Fig. 2, especially the arrangement of bottom arrows.*

**Our Response:** The authors thank the reviewer for this comment. According to the reviewer's suggestion, the Fig.2 is redrawn.

**Actions on Manuscript:** The figure2 redrawn is shown below:

**Fig 2. LSTM block model diagram.**

**Comment 4:**  *Many terms in the equation part of LSTM appear to be missing.*

**Our Response:** The authors thank the reviewer for this comment. According to the reviewer's suggestion, the equations are re-edited.

**Actions on Manuscript:**

The update calculation of Forget gate implementation is as follows:

$f_{gate}=\sigma\left( W_{f}\cdot\left[ a^{\left( t-1 \right)},x^{\left( t \right)} \right]+b_{f} \right)$	(5)

where $W_{f}$is the weight,$a^{\left(t-1\right)}$is the input of the previous cell,$x^{\left(t\right)}$is the input of the current cell,$\sigma$is the sigmoid function, and $b_{f}$is the bias. The forgetting gate reads $a^{\left(t-1\right)}$and$x^{\left(t\right)}$ and then outputs a value between 0 and 1 to the cell state $c^{\left(t-1\right)}$, where 1 indicates that information is completely retained and 0 indicates that information is completely dropped.

The in gate is used to decide how much new information is added to the current cell state, and the specific process of the in gate is expressed as

$i_{gate}=\sigma\left( W_{i}\cdot\left[ a^{\left( t-1 \right)},x^{\left( t \right)} \right]+b_{i} \right)$	(6)

$C_{t\mathrm{anh}}=\tanh\left( W_{C}\cdot\left[ a^{\left( t-1 \right)},x^{\left( t \right)} \right]+b_{C} \right)$	(7)

$C^{\left( t \right)}=f_{gate}*C^{\left( t-1 \right)}+i_{gate}*C_{tanh}$	(8)

where$W_{i}$、$W_{C}$is the weight,$b_{i}$、 $b_{C}$is the bias, the update gate first uses the sigmoid function to calculate the information to be updated, and then uses the tanh function to extract the updated content.

The output gate was used to determine the final output information of the cells. First, the sigmoid function calculates which information needs to be output, and then the tanh layer is used to output this information. The specific calculation process is as follows:

$O_{gate}=\sigma\left( W_{O}\cdot\left[ a^{\left( t-1 \right)},x^{\left( t \right)} \right]+b_{O} \right)$	(9)

$a^{\left( t \right)}=O_{gate}*\tanh\left( C^{\left( t \right)} \right)$	(10)

where$W_{O}$ is the weight, and$b_{O}$ is the bias.

**Comment 3:**  *Correct the grammatical errors.*

**Our Response:** The authors thank the reviewer for this comment. According to the reviewer's suggestion, the authors have corrected the grammatical mistakes of the recognition principle section.

**3.** **Experimental data**

**Comment 1:** *As seen in its original paper, the name of the dataset is ‘**ShipsEar’. The authors have mentioned it as ‘**SHIPSEAR’. Change it accordingly in all places.*

**Our Response:** The authors thank the reviewer for this comment. According to the reviewer's suggestion, the SHIPSEAR have been changed to the ShipsEar in all place of the manuscript.

**Comment 2:** *Use capital letters for starting a title word such as ‘Category’ and ‘Ship types’ in Table 1.*

**Our Response:** The authors thank the reviewer for this comment. According to the reviewer's suggestion, the tittle word initials of the category and ship types in Table 1 has been capitalized.

**Actions on Manuscript:** The Table 1 is shown below：

**Table1. Dataset classification.**

| **Category** | **Ship types** |
| --- | --- |
| **A** | Fishing boats; Trawlers; Mussel boats; Tugboats; Dredgers |
| **B** | Motorboats; Pilot boats; Sailboats |
| **C** | Passenger ferries |
| **D** | Ocean liner; Ro-Ro vessels |
| **E** | Background noise recordings |

**Comment 3:** *In the Data processing section, the authors need to justify why they have used the mentioned features. Also, the formula for the extraction of these features must be provided or at least they must be cited.*

**Our Response:** The authors thank the reviewer for this comment. According to the reviewer's suggestion, The Mel spectrum and MFCC used in the manuscript are commonly used features in the field of sound recognition. Converting the frequency of the audio signal to the Mel scale can better characterize the distribution features of the signal in the low frequency band, which is more in line with the human auditory. To characterize the features of audio signals more comprehensively after reviewing the relevant literature, we added the chromatogram, spectral contrast and tonnetz commonly used in music theory. We not only analyze the samples in terms of frequency, but also analyze the pitch distribution of the audio through the knowledge of music theory, so that the sample features can be extracted more completely and the recognition accuracy of underwater acoustic targets can be improved.

**Comment 4:**  *Have the authors experimented with a single feature like Mel spectrogram alone or MFCC alone? If so, what is the trend of accuracy? How the authors have come up with 5 features? Is there any feature selection method employed?*

**Our Response:** The authors thank the reviewer for this comment. According to the reviewer's suggestion, the authors have conducted experiment to the feature of the Mel spectrogram、MFCC and fusion by using T-SNE visualization. At the same time，the reason why authors came up with 5 features has been answered in the response to the comment 3 of the Experimental data section.

**Actions on Manuscript:** In the part of the experimental data, the following comments are added to enrich the manuscript and improve the quality of the paper:

To make the five fusion extraction features of the input audio express more comprehensively, we conducted T-SNE visualization of single extracted Mel spectrum, MFCCs feature and fusion feature, and the results are shown in Figure 4.

(a) Mel spectrum	(b) MFCCs

(c) Fusion feature

Fig 4. T-SNE visualization result of the Mel spectrum, MFCCs feature and fusion feature.

It can be seen from the figure that the fusion feature is more separable than the single feature, presumably because the spectral contrast and other musical theory features used can more sensitively capture the tonal features of the audio. Therefore, the subsequent research in this paper will take fusion features as input.

**Comment 5:** *It is seen that the authors have compressed the Mel spectrogram to obtain 1D data to feed to a 1D convolution layer. Have the authors experimented with 2D Mel spectrogram along with 2D CNN? If so, compare the results to justify why the proposed architecture is better.*

**Our Response:** The authors thank the reviewer for this comment. According to the reviewer's suggestion, because the dataset about ShipsEar that used in this manuscript are one-dimensional sound signals, and the three features of chromatogram, spectral contrast and tonnetz are all one-dimensional vectors, together with the Mel spectrogram and MFCCs, a total of 5 features are fused and finally as the audio data input the network. Therefore the relevant contents of feature extraction in the experimental data part of this manuscript are operated by 1D CNN.

**Comment 6:** *What is the significance of the audio serial number in Table 2? Is there any specific reason for showing it in table?*

**Our Response:** The authors thank the reviewer for this comment. According to the reviewer's suggestion, the authors have added a paragraph above Table 2 to illustrate Table 2 in the experimental data section part.

**Actions on Manuscript:** To enable reviewers and other researchers to understand the content in Table 2, an explanatory paragraph has been added above Table 2.

We manually screened the audio in accordance with the original annotation, removed some unprocessed audio with poor recording effect, and processed the remaining audio to obtain the actual data set.

To make it easier for other researchers to use the ShipsEar dataset, each audio piece in the dataset is assigned a number, and the audio serial number used is indicated in Table 2.

**Comment 7:** *In the second paragraph of Section 3.3, the authors have mentioned “The characteristics of ship…...current moment”. What is meant by that? Use proper technical words to construct such contexts.*

**Our Response:** The authors thank the reviewer for this comment. According to the reviewer's suggestion, the authors have revised the second paragraph of Section 3.3 .

**Actions on Manuscript:**To enable reviewers and other researchers to understand this part, the authors have revised the descriptive content of the second paragraph of Section 3.3 as follows:

The characteristics of ship’s underwater acoustic signal is time-varying, and we can use LSTM network to capture the characteristics of the current moment and the historical information of the previous moment.

**Comment 8:** *The direction of writing in Fig. 4 is non-standard. It is preferable to reverse the direction for improved readability (refer to such diagrams in other articles).*

**Our Response:** The authors thank the reviewer for this comment. Due to the newly added pictures before, figure 4 becomes figure5 in order, according to the reviewer's suggestion, the direction of writing in figure 5 has been revised to improve readability.

**Actions on Manuscript:** The revised figure5 is shown below:

**Fig 5. Joint network model.**

**Comment 9:**  *It is recommended to provide 1 or 2 sentences describing the overall structure of the network, such as the number of convolution layers, max pooling, LSTM and dense layers. The details of individual layer can however be provided in a table, as in Table 3.*

**Our Response:** The authors thank the reviewer for this comment. According to the reviewer's suggestion, the details of individual layer is provided in Table 3.

**Actions on Manuscript:** The Table 3 containing the details of individual layer is shown below:

**Table 3. Network parameter table.**

| **Layer type** | **Output Shape** | **Param** |
| --- | --- | --- |
| **Conv_1D** | 191×64 | 256 |
| **Maxpooling1D** | 63×64 | 0 |
| **Conv_1D** | 62×128 | 24704 |
| **Maxpooling1D** | 20×128 | 0 |
| **Dropout** | 20×128 | 0 |
| **LSTM** | 32×1 | 20608 |
| **Dropout** | 32×1 | 0 |
| **Dense** | 5×1 | 165 |

**Comment 10:** *Nothing is mentioned about the activation function used.*

**Our Response:** The authors thank the reviewer for this comment. According to the reviewer's suggestion, the content of activation function is added in Table 4 of the training result part.

**Actions on Manuscript:** The Table 4 containing the activation function is shown below:

**Table 4. Network training parameter.**

| **Parameters** | **Parameter Settings** |
| --- | --- |
| **Loss** | Categorical_crossentropy |
| **Optimizer** | Adam |
| **Metrics** | Accuracy |
| **Batch_size** | 64 |
| **Epochs** | 100 |
| **Activation function(CNN)** | ReLU |
| **Activation function(LSTM)** | Sigmoid |

**Comment 11:** *Table 3 is an exact copy of the model summary obtained with that particular deep learning framework/library. It is recommended to modify it while including in a technical paper, such as replacing ‘None’ by the corresponding batch size, naming each layer appropriately etc.*

**Our Response:** The authors thank the reviewer for this comment.According to the reviewer's comment, the ‘none’ are replaced by the corresponding batch and each layer is renamed appropriately in Table 3.

**Actions on Manuscript:** The Table 3 is shown below:

**Table 3. Network parameter table.**

| **Layer type** | **Output Shape** | **Param** |
| --- | --- | --- |
| **Conv_1D** | 191×64 | 256 |
| **Maxpooling1D** | 63×64 | 0 |
| **Conv_1D** | 62×128 | 24704 |
| **Maxpooling1D** | 20×128 | 0 |
| **Dropout** | 20×128 | 0 |
| **LSTM** | 32×1 | 20608 |
| **Dropout** | 32×1 | 0 |
| **Dense** | 5×1 | 165 |

**Comment 12:**  *Correct the grammatical mistakes here and there.*

**Our Response:** The authors thank the reviewer for this comment. According to the reviewer's suggestion, the authors have corrected the grammatical mistakes of the experimental data section.

**4.** **Experimental results**

**Comment 1:** *What is the validation data used in Fig. 5 and Fig. 6? Is it the test data itself or is it derived from the training set? It is always recommended to use separate training, validation and test sets. Provide good captions for the figures such as using ‘variation’ instead of ‘change’.*

**Our Response:** The authors thank the reviewer for this comment. Due to the newly added pictures before, figure 5 and figure 6 become figure 6 and figure 7 in order. According to the reviewer's suggestion, the authors have added a paragraph above figure6 and figure7 to illustrate the two figures.

**Actions on Manuscript:** The new addition contents and figure6、figure7 with the captions modified are as follows:

After 100 epochs, we obtained the loss and accuracy curves, as shown in Figure 6 and 7. The curve composed of blue points is the change curve of the training set, and the red curve is the test set.

**Fig 6. Variation of accuracy. Fig 7. Variation of loss.**

**Comment 2:** *The authors should modify the caption for Fig. 7.*

**Our Response:** The authors thank the reviewer for this comment. Due to the newly added pictures before, figure 7 becomes Figure 8 in order. According to the reviewer's suggestion, the authors have modified the caption of figure 8.

**Actions on Manuscript:** Figure 8 with the caption modified is shown below:

**(a) LTSM	(b) 1D-CNN	(c) Joint Network**

**Fig 8. Confusion matrices for three networks**

**Comment 3:** *Are figures 8, 9 and 10 actually needed? Sufficient information is already available in Fig. 7. Hence, Fig. 8-10 appear to be redundant and space consuming. The authors may provide other relevant performance metrics instead of these.*

**Our Response:** The authors thank the reviewer for this comment. Due to the newly added pictures before, figure 8、figure 9、figure10 become figure 9、figure 10 and figure 11 in order. According to the reviewer's suggestion, the authors have removed figure 9、figure 10 and figure 11.

**Comment 4:** *It is better to use P-R curves instead of Table 7 in order to facilitate easy and appealing comparison of the three networks.*

**Our Response:** The authors thank the reviewer for this comment. According to the reviewer's suggestion, P-R curves are often used for two-class classification, and our manuscript is for five-class classification. So we use histograms to make the comparison of the three networks easy and appealing.

**Actions on Manuscript:** The three histograms added in the experimental results section are shown below：

**Fig9. The result of precision about different categories.**

**Fig 10. The result of recall about different categories.**

**Fig 11. The result of recall about different categories.**

**Comment 5:** *Use appropriate technical words in the paragraph starting with “In order to* *prevent the experimental...”. The authors may use “to ensure repeatability” like that.*

**Our Response:** The authors thank the reviewer for this comment. According to the reviewer's suggestion, the author revised the description about the paragraph starting with“In order to prevent the experimental...”.

**Actions on Manuscript:** The revised paragraph reads as follows：

To ensure repeatability, we conducted 30 training sessions for the three network models, and compared the network recognition accuracy after 30 training sessions. The 30 training results for the three networks are shown in Figure 12.

**Comment 6:** *After Fig. 11, the authors have mentioned that the joint network is performing well. They must also write an inference on why some classes have low recognition.*

**Our Response:** The authors thank the reviewer for this comment. Due to the newly added pictures before, figure 11 becomes figure 12 in order. According to the reviewer's suggestion, the author have added an inference on why some classes have low recognition.

**Actions on Manuscript:** Inferences with low recognition rates for some classes are shown below:

However, we can also see that the network does not have a very good recognition effect for Type B, and this is presumed to be caused by the insufficient training of the network owing to the small number of type B samples. Class E, with the same small number of samples is environmental noise, which is clearly differentiated from other categories; thus, the recognition effect is good.

**Comment 7:** *Table 8 is absolutely not needed. It the authors want to show the variations among different trials for the three networks, they may use a box plot.*

**Our Response:** The authors thank the reviewer for this comment. According to the reviewer's suggestion, the authors have removed table 8.

**Comment 8:**  *Correct the sentence construction and grammatical errors.*

**Our Response:** The authors thank the reviewer for this comment. According to the reviewer's suggestion, the authors have corrected the sentence construction and grammatical errors of the experimental results section.

**5.** **Conclusion**

**Comment 1:**  *The first sentence is poorly written. Reframe it.*

**Our Response:** The authors thank the reviewer for this comment. According to the reviewer's suggestion, the authors have reframed the first sentence of the conclusion section.

**Actions on Manuscript**: The first sentence of the conclusion section reframed is as follows:

In this study, a new network structure combining a 1D CNN and LSTM network is proposed and applied to underwater acoustic target recognition. The joint network can combine the advantages of the two neural networks to extract features from input data more comprehensively.

**Comment 2:** *Modify conclusion to include certain promising values of the experimental results.*

**Our Response:** The authors thank the reviewer for this comment. According to the reviewer's suggestion, the authors have illustrated the value of the experimental results in the second paragraph of the conclusion section.

**Actions on Manuscript**: The value of the experimental results are as follows:

The experimental results using the ShipsEar underwater vessel dataset show that the proposed joint network has a higher recognition accuracy than traditional neural networks, and the network has a simple structure, fewer parameters, and shorter training time. This provides a new development direction for underwater acoustic target recognition methods.

**Comment 3:** *It is mentioned that the dataset used is single. What is meant by that? Reframe it for better understanding.*

**Our Response:** The authors thank the reviewer for this comment. According to the reviewer's suggestion, the authors have reframed “the dataset used is single”for better understanding.

**Actions on Manuscript**: The reframed sentence is as follows:

The limitation of this study is that only one dataset is used in experimentation. Both the training and test sets originate from the ShipsEar dataset.

**For Reviewer: 2**

**Response to the comments**

**Comment 1:** *The authors are suggested to carefully explain the values of parameters related with neural networks used in simulation.*

**Our Response:** The authors thank the reviewer for this comment. According to the reviewer's suggestion, the authors have explained the values of parameters related with neural networks used in simulation as follow:

（1）The input feature dimension is (192,1), then enter the convolutional layer 1, the size of the convolutional kernel in the convolutional layer 1 is 3*1, the stride is 1, and the output dimension is (191,64). “191”is the result of the convolution kernel sliding 191 times on the input vector and doing 191 operations;“64”is the number of the filters we set to extract features.“(191,64)”is obtained by performing convolution operation on the input (192,1) with 64 convolution kernels respectively.

（2）The pooling layer 1: select the maximum pooling, and the pooling size is 3，that is, select the largest one in every three data as the output,191/3=63.6.., Remove the digits after the decimal point and keep only integers, get 63. Then the“(63,64)”is obtained by performing operation like the convolutional layer 1.

（3）The convolutional layer 2: The size of the convolutional kernel is 1, “128”is the number of the filters we set to extract features.“(62,128)”is obtained by performing by performing operation like the convolutional layer 1.

（4）The pooling layer 2: Select the maximum pooling, and the pooling size is 3，that is, select the largest one in every three data as the output,62/3=20.6.., Remove the digits after the decimal point and keep only integers, get 20. Then the“(20,128)”is obtained by performing operation like the convolutional layer 2.

（5）Dropout：The dropout of the CNN does not change the size of the output shape.

（6）LSTM:“32”is the number of the units we set in the LSTM，and the output dimension is（32,1）.

（7）Dropout：The dropout of the LSTM does not change the size of the output shape.

（8）Dense：The Dense layer maps the extracted features to the output space to achieve five classifications, so the output dimension is (5,1).

**Comment 2:** *The authors discuss the main structure of neural networks used in this paper, such as CNN and LSTM. However, these two structures are the main popular neural networks widely used in different applications. Therefore, the authors are suggested to highlight the main contributions of this paper and differences between the current work and some published works.*

**Our Response:** The authors thank the reviewer for this comment. According to the reviewer's suggestion, the authors have explained the reason why CNN and LSTM are used to build a joint network as follows：

1D CNN is widely used in speech fields such as sequence modeling and natural language processing, while long short-term memory (LSTM) is suitable for processing and predicting time series. However, the recognition framework of a single neural network makes the extraction of all features of underwater acoustic signals challenging, the research direction usually focused on the development of deeper and more complex networks. Therefore, building a new network model by combining various network structures is a good solution. That is the reason why authors choose 1D CNN and LSTM to build joint network for underwater acoustic target recognition.

**Comment 3:** *The English wording and grammar should be carefully polished to mitigate misunderstanding.*

**Our Response:** The authors thank the reviewer for this comment. According to the reviewer's suggestion, the authors have reorganized and proofread the entire manuscript, the wording and grammar are corrected to ensure that the manuscript is easy to understand.
