# Supplementary material for: Underwater acoustic target recognition method based on a joint neural network
Source: PLoS One. 2022 Apr 29;17(4):e0266425. doi: 10.1371/journal.pone.0266425 (PMC9053803; doi:10.1371/journal.pone.0266425)
Supplement: Attachment — Submitted filename: Response to Reviewers.docx [file pone.0266425.s002.docx]

**Paper Title:** Underwater Acoustic Target Recognition Method Based on

Joint Neural Network

**Authors:** Xingcheng Han, Chenxi Ren, Liming Wang, Yunjiao Bai

**Manuscript Number:** PONE-D-21-38631	R1

**Date:** March 3, 2022

**Response to the Reviewers**

We would like to thank the reviewers and the editor for their time in reviewing our paper and suggestions for improving it. In this document we have written a detailed point-by-point response to the reviewers to address their comments and explain the corresponding changes made in the manuscript. Throughout this document, we use the following conventions:

- *Blue text in Italics* = Reviewers’ comments.
- Red text = New additions to the manuscript.
- Black text = Authors’ responses.

This response letter is a self-contained document that conveniently includes all the revisions.

We hope that the current revision meets your approval.

Kind regards,

Xingcheng Han, Chenxi Ren, Liming Wang, Yunjiao Bai

We would like to thank the reviewers for their valuable comments, which have helped us to further improve the contribution of the paper. We have addressed all the comments and suggestions and revised our manuscript as follows:

**For Reviewer: 1**

**Response to the comments**

**Comment 1:** *For tables 1 to 5, it will be visually more appealing to use the same format* *used in the originally submitted manuscript, i.e. use borders only for title and at the bottom than using borders everywhere.*

**Our Response:** The authors thank the reviewer for this comment. According to the reviewer's suggestion, tables 1 to 5 have been revised, the formats are same with the originally submitted manuscript.

**Comment 2:** *Some paragraphs have only one or two lines. Merge paragraphs that are too short.*

**Our Response:** The authors thank the reviewer for this comment. According to the reviewer's suggestion, the authors have merged the paragraphs with only one or two lines.

**Abstract：**

**Comment 1:** *It is more common to use ‘indices’ instead of ‘indexes’.*

**Our Response:** The authors thank the reviewer for this comment. According to the reviewer's suggestion, the word ‘indexes’ has been replaced by word ‘indices’ in abstract section.

**Introduction：**
**Comment 1:** *Paragraph 4: In the second line, the continuity for LSTM has been lost; Reframe it.*

**Our Response:** The authors thank the reviewer for this comment. According to the reviewer's suggestion, the authors have re-described the LSTM structure.

**Actions on Manuscript:** In the paragraph 4 of the introduction, the following sentences have been added to improve the quality of the paper:

The long short-term memory (LSTM) structure can not only capture the characteristics of the sequence itself, but also take into account the continuity of the sequence.

**Comment 2:** *Paragraph 5 & 6: Grammatical errors- ‘seem’, ‘reduce’ and ‘build’ are used instead of ‘seems’, ‘reduces’ and ‘built’ respectively.*

**Our Response:** The authors thank the reviewer for this comment. According to the reviewer's suggestion, three words“‘seem’ 、‘reduce’、 ‘build’”have been replaced by “‘seems’ 、‘reduces’、 ‘built’”in paragraph 5 & 6 of the introduction section.

**Recognition principle：**

**Convolutional neural network:**

**Comment 1:**  *Technical mistake in the line: “Finally, the predicted category labels can be obtained through the convolution layers”.*

**Our Response:** The authors thank the reviewer for this comment. According to the reviewer's suggestion, the authors have modified this sentence. The specific changes are as follows: “Finally, the predicted category labels can be obtained through the output layer.”

**Comment 2:** *Paragraph 3: Comma should be provided after respectively; instead it is provided before.*

**Our Response:** The authors thank the reviewer for this comment. According to the reviewer's suggestion, comma has been provided after the word "respectively".

**Long short-term memory:**

**Comment 1:** *Paragraph 3: ‘Forgetting gate’ is provided instead of ‘Forget gate’.*

**Our Response:** The authors thank the reviewer for this comment. According to the reviewer's suggestion, the ‘Forget gate’ has been replaced by ‘Forgetting gate’ in Paragraph 3.

**Comment 2:**  *Incorrect commas between Wi & Wc and between bi & bc, after equation 8.*

**Our Response:** The authors thank the reviewer for this comment. According to the reviewer's suggestion, the authors have removed the commas between Wi & Wc and between bi & bc, after equation 8.At the same time，the authors have modified this sentence.

**Actions on Manuscript:** After equation 8，the following sentences have been added to improve the quality of the paper:“where $W_{i}$ and $W_{C}$ are the weight, $b_{i}$ and $b_{C}$ are the bias.”

**Data processing：**

**Comment 1:** *Paragraph 4: Spelling mistake - ‘calcultated’.*

**Our Response:** The authors thank the reviewer for this comment. According to the reviewer's suggestion, the authors have re-spelled the word, and the wrong word ‘calcultated’ has been replaced by ‘calculated’ in paragraph 4.

**Comment 2:**  *The authors need not elaborate much on audio related theories behind* *spectral contrast and tonnetz. There is no need to mention the* *librosa function used to calculate tonnetz; instead provide* *equations to extract these features, if available. The use of audio everywhere does not seem a good practice in an underwater target recognition paper, since an audio signal is mostly associated with human hearing and spans frequencies from 20 Hz - 20 kHz, which is not the scenario of a ship signal. The authors may instead mention that they have used features that are mostly auditory-inspired and in other places where they have mentioned audio signals, they may either use acoustic signals or target signals (even in Fig 3.)*

**Our Response:** The authors thank the reviewer for this comment. According to the reviewer's suggestion, the audio related theories behind spectral contrast and tonnetz and the librosa function used to calculate tonnetz has been removed. Since the authors did not find specific equations, so the equations can’t be added in the paper. At the same time, according to the reviewer's suggestion，the author have revised ‘audio signal’ in paper, the ‘audio signals’ have been replaced by ‘acoustic signals’.

**Comment 3:** *Paragraph 9: Grammatical mistake in the line “each audio data will obtain a (192,1) feature vector”.*

**Our Response:** The authors thank the reviewer for this comment. According to the reviewer's suggestion, the authors have modified the sentence，the specific changes are as follows: “each acoustic signal will extract a feature vector with a dimension of (192,1).”

**Comment 4:** *It is suggested to change the spelling of T-SNE to t-SNE in multiple places.*

**Our Response:** The authors thank the reviewer for this comment. According to the reviewer's suggestion, the word‘T-SNE’has been replaced by‘t-SNE’in paper.

**Comment 5:** *Wrong title for Figure 4 wrong - t-SNE embedding of digits?*

**Our Response:** The authors thank the reviewer for this comment. According to the reviewer's suggestion, the author have modified the title of figure 4. The modified pictures are as follows：

（a）Mel spectrum	(b) MFCCs	(c) Fusion feature

Fig 4. t-SNE visualization result of the Mel spectrum, MFCCs feature and fusion feature.

**Comment 6:** *Provide legends in Figure 4 to understand the various categories.*

**Our Response:** The authors thank the reviewer for this comment. According to the reviewer's suggestion, the legends have been provided in figure 4.The modified pictures are as follows：

（a）Mel spectrum	(b) MFCCs	(c) Fusion feature

Fig 4. t-SNE visualization result of the Mel spectrum, MFCCs feature and fusion feature.

**Network Construction:**

**Comment 1:** *Paragraph 3: Why 'DENSE' is provided in capital letters?*

**Our Response:** The authors thank the reviewer for this comment. According to the reviewer's suggestion, the author have modified the word‘DENSE’, and the word‘DENSE’ has been replaced by‘dense’.

**Training results:**

**Comment 1:** *Paragraph 3: Incorrect sentence construction - “To verify whether the performance of the combined network proposed in this study…….”.*

**Our Response:** The authors thank the reviewer for this comment. According to the reviewer's suggestion, the authors have modified this sentence，the specific changes are as follows: “In order to verify the performance of the joint network proposed in this study, we compared its recognition accuracy with 1D-CNN and LSTM networks, and the results are shown in Table 5.”

**Comment 2:** *Redundancy in the word ‘category’ just before Fig 9.: “For each category, the precision, recall and F1 score for this category…”.*

**Our Response:** The authors thank the reviewer for this comment. According to the reviewer's suggestion, the authors have modified this sentence，the specific changes are as follows: “For each category, the precision, recall and F1 score were calculated.”

**Comment 3:** *Why it is mentioned “The result of the precision…” in Fig 9, “The result of the recall…” in Fig. 10 and “The result of F1 score…” in Fig. 11? Precision, recall and F1-score themselves are results. Simply write Precision, Recall and F1-score for the figure titles & captions. Provide a label such as ‘category’ for x-axis.*

**Our Response:** The authors thank the reviewer for this comment. According to the reviewer's suggestion, the author have modified the title of figure 9、figure 10 and figure 11. The modified pictures are as follows：

**Fig9. Precision about different categories. Fig 10. Recall about different categories.**

**Fig 11. F1 Score about different categories.**

**Comment 4:**  *The caption for Fig. 11 is wrongly provided.*

**Our Response:** The authors thank the reviewer for this comment. According to the reviewer's suggestion, the author have modified the caption of figure 11. The modified picture is as follows：

**Fig 11. F1 Score about different categories.**

**Comment 5:** *Grammatical mistake in the paragraph after Fig. 11: “evaluating the network in terms of the F1 score it is more comprehensive”.*

**Our Response:** The authors thank the reviewer for this comment. According to the reviewer's suggestion, the authors have modified the sentence，the specific changes are as follows:“it is more comprehensive to evaluate the network with the F1 score.”

**Conclusion:**

**Comment 1:** *It was suggested to include certain promising values of experimental results, but it is still not incorporated. For this, the authors may write a sentence with the* *values of accuracy, P, R and F1 of the proposed joint network, compared against LSTM and 1D CNN.*

**Our Response:** The authors thank the reviewer for this comment. According to the reviewer's suggestion, the authors have added a sentence to describe the comparison results of accuracy, P, R, and F1 of the three networks. The added sentence is as follows: “compared with 1D CNN and LSTM network, the joint neural network has higher accuracy, precision, recall and F1 score.”

**Comment 2:** *Paragraph 3: Grammatical mistake - “performance of this network was not be verified in an actual marine environment…”.*

**Our Response:** The authors thank the reviewer for this comment. According to the reviewer's suggestion, the authors have modified this sentence，the specific change is as follows:“thus the performance of this network has not been verified in an actual marine environment.”

**Comment 3:**  *Last sentence is too long and incorrectly constructed. Split the sentence instead of using so much ‘and’.*

**Our Response:** The authors thank the reviewer for this comment. According to the reviewer's suggestion, the authors have modified the sentence，the specific changes are as follows:“Our research direction is to expand the data set, collect more measured ship noise acoustic signals, optimize network parameters by increasing the number of data sets and continuous training, so as to enhance the universality of the network.”

**Reference:**

**Comment 1:**  *Reference [30] still shows “SHIPSEAR” instead of “ShipsEar”.*

**Our Response:** The authors thank the reviewer for this comment. According to the reviewer's suggestion, the word‘SHIPSEAR’has been replaced by‘ShipsEar’in Reference [30].
